# High-sensitivity C-reactive protein to lymphocyte ratio as a novel biomarker for predicting postoperative delirium in elderly patients with hip fractures: A retrospective cohort study

**DOI:** 10.1097/MD.0000000000047650

**Published:** 2026-02-06

**Authors:** Song Liu, Shanshan Zhang, Xiuting Li, Yanbin Zhu, Yahui Zhang

**Affiliations:** a Department of Orthopaedic Surgery, Hebei Medical University Third Hospital, Shijiazhuang, Hebei, China; b Department of anesthesiology, Hebei Medical University Third Hospital, Shijiazhuang, Hebei, China.

**Keywords:** Delirium, Hip fracture, Immune, Inflammatory, Risk prediction model

## Abstract

Emerging evidence suggests that inflammatory and immune biomarkers may serve as prognostic indicators across diverse clinical contexts. This study sought to investigate the association between the high-sensitivity C-reactive protein to lymphocyte ratio (hs-CLR) and the risk of postoperative delirium in elderly patients undergoing surgery for hip fractures. We conducted a retrospective cohort study of patients aged 60 years or older who underwent surgical management for hip fractures at a university-affiliated tertiary hospital between January 2021 and December 2024. The primary exposure was hs-CLR, and the primary endpoint was 30-day postoperative delirium, ascertained through systematic review of inpatient medical records and confirmed by outpatient and telephone follow-up. The relationship between hs-CLR and delirium was explored using restricted cubic spline modeling, receiver operating characteristic curve analysis, univariate comparisons, and multivariate logistic regression. A total of 582 patients (192 males, 390 females; mean age 73.8 ± 8.4 years) were included. The 30-day incidence of delirium was 21.3% (124/582). Both unadjusted and adjusted restricted cubic spline analyses revealed a significant nonlinear dose-response association between hs-CLR and delirium risk (*P* <.05). Using a clinically determined threshold of 36.0, patients were stratified into low and high hs-CLR groups. Patients with elevated hs-CLR were more often male, had a higher comorbidity burden (reflected by higher age-adjusted Charlson comorbidity index and American Society of Anesthesiologists class) and poorer nutritional and general health status (lower serum albumin, red blood cell count, and serum sodium) than those with low hs-CLR. Multivariable logistic regression demonstrated that hs-CLR ≥36.0 was independently associated with increased odds of postoperative delirium (fully adjusted model: odds ratio 2.35, 95% CI 1.47–3.74; backward elimination model: odds ratio 2.28, 95% CI 1.46–3.54). This study delineates a robust nonlinear association between elevated hs-CLR and the risk of postoperative delirium in elderly hip fracture patients, and establishes that hs-CLR ≥36.0 is independently associated with delirium. These findings underscore the potential value of incorporating hs-CLR into preoperative risk stratification to enhance perioperative management in this vulnerable population.

## 1. Introduction

Delirium is an acute, reversible disturbance of attention, cognition, and consciousness level, with postoperative incidences ranging from 14% to 61% in elderly patients following hip fracture surgery.^[1,2]^ This condition can lead to significant morbidity, increased mortality, a higher risk of joint dislocation, and prolonged hospitalization.^[3]^ Although effective treatment options remain limited, it is estimated that approximately one-third of delirium cases are preventable,^[4]^ underscoring the need for early assessment and identification of high-risk patients.

The mechanisms underlying postoperative delirium following hip fracture remain poorly understood. Recent studies suggest a potential association with systemic inflammatory responses, indicating that the inflammatory marker levels may correlate with the delirium severity.^[5]^ Elevated inflammatory markers, such as interleukin-6 and tumor necrosis factor-alpha (TNF-α), have been observed among patients with delirium, suggesting their potential utility as predictive biomarkers.^[6,7]^ However, the complexity and cost of measuring these inflammatory markers limit their use in routine clinical practice. In contrast, high-sensitivityC-reactive protein (hs-CRP) is an inexpensive and effective biomarker for assessing general levels of inflammation, and it has been associated with the risk of stroke and other neuropsychiatric conditions.^[8]^ Additionally, delirium following hip fracture has also been associated with impaired negative immunoregulatory mechanisms.^[9]^ Recent research using single-cell sequencing and Mendelian randomization has shown that only lymphocyte and B cell counts are causally associated with delirium risk.^[10]^ Thus, theoretically, the hs-CRP lymphocyte ratio (hs-CLR), may represent a promising predictor of postoperative delirium in elderly hip fractures, potentially enhancing the predictive capabilities of both original biomarkers when combined. Although hs-CLR has demonstrated predictive value for various adverse events in diverse clinical settings,^[11–14]^ data regarding its association with postoperative delirium remain limited and are often constrained by small sample sizes and inadequate control for confounding.

Therefore, the present study aimed to investigate the association between preoperative hs-CLR and postoperative delirium (POD) in elderly patients with hip fractures. We hypothesized a priori that higher preoperative hs-CLR would be independently associated with an increased risk of POD and would improve delirium risk discrimination beyond established clinical predictors.

## 2. Methods

This study was a retrospective single-center analysis. Prior to its commencement, the study protocol was approved by the ethics committee of the Hebei Medical University Third Hospital (No. W2020-012-1), a tertiary care facility designated as a Level Ⅰ trauma center, serving a population of over 20 million residents. Due to the retrospective nature of the research and the anonymization of patient data, the requirement for informed consent was waived. The study followed the principles outlined in the Helsinki Declaration.

Two trained researchers conducted a thorough review of inpatient medical records to identify potentially eligible patients aged 60 years or older who had a discharge diagnosis of hip fracture and undergone surgical intervention at our institution between January 2020 and December 2024. The exclusion criteria included: high-impact mechanism (such as falls from heights greater than standing, or traffic accident); concurrent fractures or bilateral hip fractures; pathological or metastatic fracture; surgeries performed more than 21 days post-injury; existing systemic infectious conditions; blood transfusions administered prior to surgery; use of glucocorticoids or immunosuppressive therapy within 3 months preceding the injury; documentation of preoperative delirium; and incomplete follow-up data at 1 month post-surgery.

### 2.1. Exposure and outcome

Laboratory blood sampling and testing were typically conducted on the morning following admission, in accordance with the manufacturers’ protocols. The hs-CLR was calculated by dividing hs-CRP (mg/L) by lymphocyte counts (*10^9^/L). in cases where patients had multiple measurements prior to surgery, the value closest to the date of admission was selected for analysis.

The outcome was the incidence of postoperative delirium, diagnosed using the mini-mental state examination.^[15]^ The mini-mental state examination is a 30-point assessment instrument designed to assess cognitive impairment across 5 domains: orientation (10 points), registration (3 points), attention and calculation (5 points), recall (3 points), and language (9 points). The criteria for diagnosing delirium are influenced by the individual’s educational background, and thus, for elderly individuals, a score of <17 points indicates delirium if they are illiterate, <20 points if they have completed elementary school, and <24 points for those with secondary education or higher.

Patients were evaluated once daily during hospitalization from postoperative day 1 until discharge. Delirium cases were then ascertained through these in-hospital assessments, a comprehensive review of inpatient medical records for documented diagnoses or relevant notes, and telephone or outpatient follow-up at 1 month after surgery to identify any additional episodes occurring within 30 days postoperatively.

### 2.2. Covariables of interest

Covariates were selected based on previous literature, as well as clinical relevance and empirical considerations. These included demographics (age, sex, body mass index, residency), comorbidities (hypertension, diabetes mellitus, heart disease, cerebrovascular disease, chronic obstructive pulmonary disease, liver disease, renal insufficiency, peripheral vascular disease, rheumatoid disorders, active malignancy, age-adjusted Charlson comorbidity index [aCCI]), smoking status, surgery-related variables (American Society of Anesthesiologists classification, fracture location, sidedness, time to surgery, anesthesia technique, operative method, surgical duration, intraoperative bleeding, blood transfusion, bone graft use, treatment surgeon experience), and laboratory testing results (albumin, fasting blood glucose, platelet count, creatine level, red blood cell count, white blood cell count, sodium and potassium concentration.

### 2.3. Statistical analysis

To visually explore the dose-response relationship between hs-CLR and the risk of postoperative delirium, a restricted cubic spline (RCS) model with 4 knots was fitted, adjusting for all covariates. A *P*-value of <.05 was considered indicative of a statistically significant nonlinear relationship. The hs-CLR level corresponding to an odds ratio (OR) of 1.0 served as the reference point for baseline complication risk. Receiver operating characteristic (ROC) curve analysis was performed to determine the optimal cutoff value of hs-CLR for predicting delirium, maximizing the Youden index (sensitivity + specificity – 1). Based on the reference point and the ROC-generated cutoff value, the clinically relevant cutoff value was selected and accordingly patients were categorized into low and high hs-CLR groups.

Patients with low and high hs-CLR were compared univariately, using Student *t*-test or Mann–Whitney test for continuous variates, and Chi-square test or Fisher exact test for categorical variables, as appropriate. Variable with a univariate *P*-value of <.10 were incorporated into a subsequent multivariate logistic regression model. Additionally, age, sex and other variables recognized in the literature as influencing the neuropsychiatric disorders were incorporated in the model, regardless of their univariate statistical significance. Two approaches were utilized for the multivariate analysis: the “enter” selection method, which represents a fully adjusted model, and the stepwise backward elimination technique. The strength of the associations was quantified using OR and 95% confidence intervals. The goodness-of-fit for the multivariate model was assessed via the Hosmer–Lemeshow (H–L) test, and a *P*-value >.05 indicated an acceptable fit. The statistical significance level was set as *P* <.05.

Missing data were examined for all variables. The overall proportion of missing values was low (all <5% for the variables included in the analyses). Therefore, a complete-case analysis was performed, and patients with missing data for any of the relevant variables were excluded from the corresponding analyses.

Statistical analyses were conducted using SPSS 27.0 (IBM, Armonk).

## 3. Results

**Figure 1.**
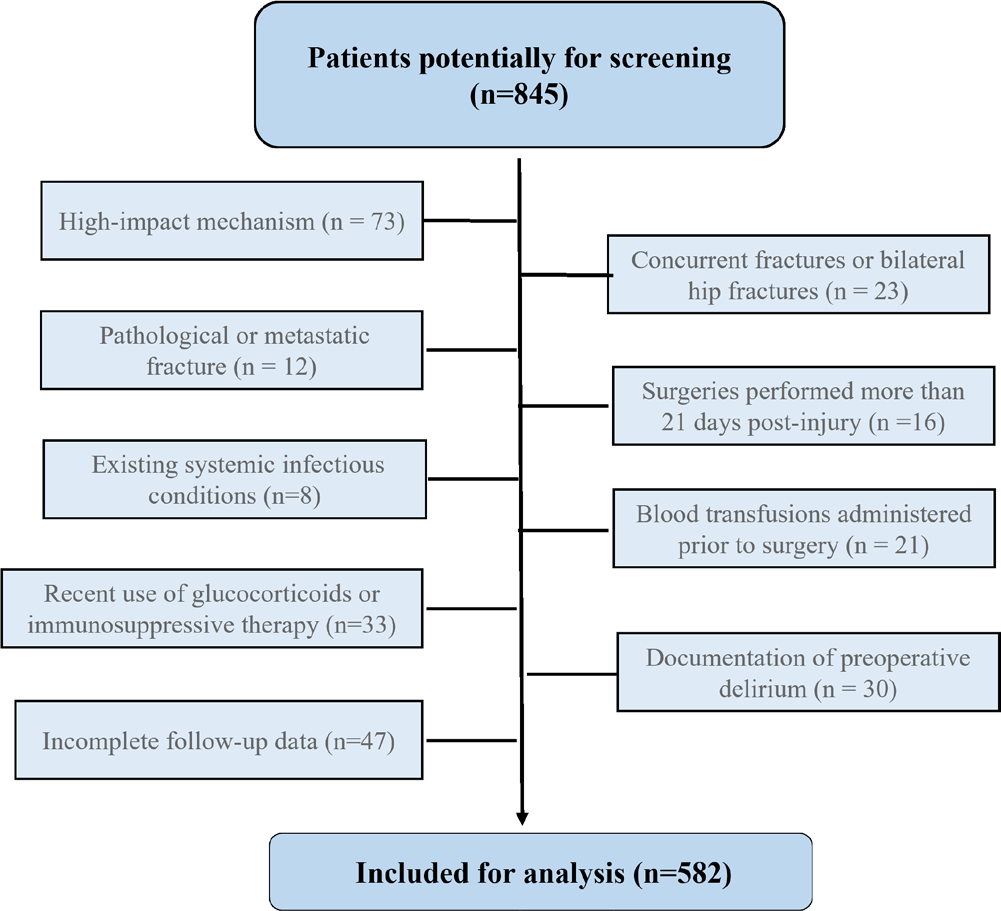
Flow chart of patient selection for the study. A total of 845 patients aged ≥65 yr who underwent surgery for hip fracture. Patients were excluded for the high-impact mechanism of injury, pathological or metastatic fracture, existing systemic infectious conditions, recent use of glucocorticoids or immunosuppressive therapy, incomplete follow-up data, concurrent fractures or bilateral hip fracture, surgery performed more than 21 d after injury, blood transfusions administered prior to surgery, or documentation of preoperative delirium. The remaining 582 patients were included in the final analysis.

A total of 582 patients were included in the analysis (see flow diagram in Fig 1), comprising 192 males (33.0%) and 390 females (67.0%), with a mean age of 73.8 ± 8.4 years (range, 60 to 101 years). Postoperative delirium was observed in 124 patients, resulting in an incidence rate of 21.3%.

**Figure 2.**
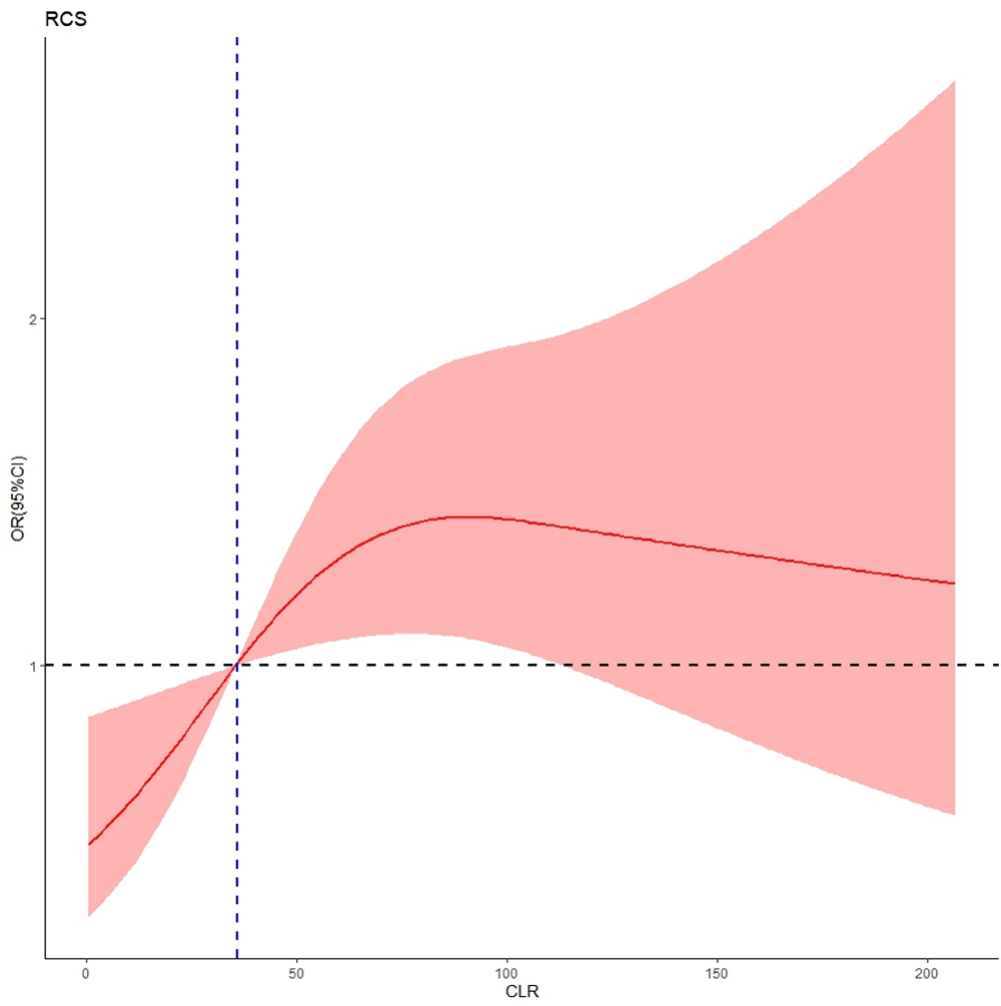
RCS curve adjusted for all variables showing the significant nonlinear relationship between hs-CLR and delirium, with baseline risk point at hs-CLR of 36.0 and ceiling risk effect at 86.0, in patients with hip fractures (n = 582). The solid red line represents OR for POD, the pink shaded area represents 95% CI and the blue vertical dashed line indicates the reference value of hs-CLR (36.0), and the black horizontal dashed line corresponds to an OR of 1.0. CI = confidence interval, POD = postoperative delirium, RCS = restricted cubic spline, hs-CLR = high-sensitivity C-reactive protein to lymphocyte ratio, OR = odds ratio.

**Figure 3.**
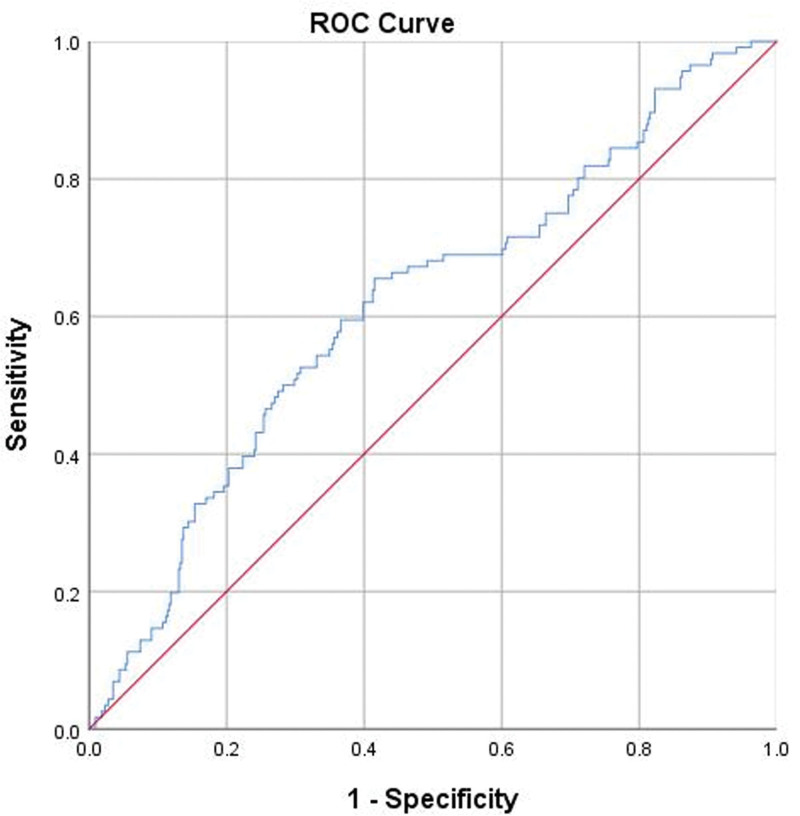
ROC curve identifying the optimal cutoff value for hs-CLR as 35.5, corresponding to a sensitivity of 0.581 and a specificity of 0.618. The area under the curve was 0.617 (*P* <.001). The blue line represents the ROC curve for hs-CLR, and the red diagonal line indicates the line of no discrimination. AUC = area under the curve, CI = confidence interval, hs-CLR = high-sensitivity C-reactive protein to lymphocyte ratio, ROC = receiver operating characteristic.

The average hs-CRP level was 48.0 ± 43.4 mg/L, while the mean lymphocyte count was 1.2 ± 0.6 *10^9^/L, yielding an hs-CLR of 51.4 ± 62.7 (range, 0.06–651.4). As depicted in Figure 2 and Figure S1, Supplemental Digital Content, https://links.lww.com/MD/R351 both unadjusted and adjusted RCS models demonstrated a significant nonlinear relationship (*P* = .001 and 0.029, respectively), indicating that as hs-CLR values increase, the risk of delirium rises until reaching a plateau at hs-CLR of 86.0, beyond which the risk remains relatively constant. The reference point for hs-CLR corresponding to an OR of 1.0, was identified as 36.0. ROC curve analysis established the cutoff value for hs-CLR to be 35.5, providing a sensitivity of 0.581 and a specificity of 0.618, with an area under the curve of 0.617 (95% CI 0.559–0.675; *P* <.001) (Fig. 3).

The hs-CLR of 36.0 was designated as the clinically relevant cutoff value, categorizing patients into low (<36.0, n = 335) and high hs-CLR (≥36.0 n = 247) groups. The unadjusted OR for delirium in the high hs-CLR group relative to the low hs-CLR group was 2.24 (95% CI 1.50–3.35).

As summarized in Table 1, the 2 groups exhibited significant differences in sex distribution (*P* = .006), aCCI (*P* = .004), American Society of Anesthesiologists classification (*P* = .045), serum albumin (*P* <.001), red blood cell count (*P* <.001), sodium concentration (*P* <.001). There was a trend towards a higher proportion of intertrochanteric fracture in patients with CLR ≥36.0 (*P* = .068).

**Table 1:** Comparison of demographic and clinical characteristics between high- and low-CLR group.

Variables	CLR <36.0 (n = 335)	CLR ≥36.0 (n = 247)	*P*
Age (year)	73.3 ± 8.4	74.4 ± 8.4	.127
Sex (male)	95 (28.4)	97 (39.3)	.006
Residency (rural)	174 (51.9)	133 (53.8)	.649
BMI (kg/m^2^)	23.4 ± 3.3	23.6 ± 3.8	.500
<18.5	32 (9.6)	16 (6.5)	.406
18.5–23.9	159 (47.5)	127 (51.4)	–
24.0–27.9	120 (35.8)	82 (33.2)	–
≥28.0	24 (7.2)	22 (8.9)	–
aCCI			
≤3	94 (28.1)	48 (19.4)	.004
4	142 (42.4)	102 (41.3)	
5	46 (13.7)	60 (24.3)	
≥6	53 (15.8)	37 (15.0)	
Hypertension	167 (49.9)	136 (55.1)	.214
Diabetes mellitus	61 (18.2)	54 (21.9)	.274
Heart disease	104 (31.0)	74 (30.0)	.779
Cerebrovascular disease	84 (25.1)	72 (29.1)	.273
COPD	28 (8.4)	30 (12.1)	.132
Liver disease	10 (3.0)	7 (2.8)	.915
Smoking			
Current	36 (10.7)	30 (12.1)	.820
Never	278 (83.0)	200 (81.0)	
Former	21 (6.3)	17 (6.9)	
Peripheral vascular disease	21 (6.3)	23 (9.3)	.170
Rheumatoid disorders			
Malignancy	12 (3.6)	4 (1.6)	.152
Renal insufficiency	15 (4.5)	17 (6.9)	.208
ASA classification			
I	15 (4.5)	17 (6.9)	.022
II	191 (57.0)	113 (45.7)	
III or more	129 (38.5)	117 (47.4)	
Fracture location			
Intertrochanteric	132 (39.4)	116 (47.0)	.068
Femoral neck	203 (60.6)	131 (53.0)	
Sidedness			
Left	173 (51.6)	129 (52.2)	.889
Right	162 (48.4)	118 (47.8)	
Time from injury to operation (h)			
≤ 72	236 (70.4)	169 (68.4)	.599
> 72	99 (29.6)	78 (31.6)	
Anesthesia technique (general)	148 (44.2)	118 (47.8)	0.390
Operative method			
Arthroplasty	94 (28.1)	70 (28.3)	0.941
Fracture repair	241 (71.9)	177 (71.7)	
Surgical duration (min)			
< 120	240 (71.6)	172 (69.6)	.499
≥ 120	95 (28.4)	75 (30.4)	
Intraoperative bleeding (mL)			
< 200	181 (54.0)	130 (52.6)	.165
200–399	91 (27.2)	82 (33.2)	
≥ 400	63 (18.8)	35 (14.2)	
Intraoperative allogeneic blood transfusion	53 (15.8)	51 (20.6)	.133
Allogeneic bone graft	11 (3.3)	12 (4.9)	.335
Surgeon seniority (≥ 10 years)	313 (93.4)	235 (95.1)	.385
Serum albumin (g/L)	35.7 ± 6.1	32.9 ± 4.7	<.001
FBG (mmol/L)	6.53 ± 1.93	6.66 ± 1.94	.432
Platelet count (*10^12^/L)	220.5 ± 87.6	210.5 ± 74.2	.147
Creatine (µmol/L)	65.5 ± 51.9	61.8 ± 18.8	.280
RBC count (*10^12^/L)	3.75 ± 0.59	3.49 ± 0.54	<.001
WBC count (*10^9^/L)	8.8 ± 2.9	9.0 ± 2.9	.519
Sodium concentration (mmol/L)	138.1 ± 3.8	136.9 ± 3.6	<.001
Potassium concentration (mmol/L)	4.02 ± 0.52	4.00 ± 0.46	.624

aCCI = age-adjusted Charlson comorbidity index, ASA = American Society of Anesthesiologists, BMI = body mass index, CLR = high-sensitive C-creative protein lymphocyte ratio, COPD = chronic obstructive pulmonary disease, FBG = fasting blood glucose, PLR = platelet lymphocyte ratio, RBC = red blood cell.

Both the fully adjusted model and backward selection model revealed a significant association between high hs-CLR and an increased risk of delirium, with ORs of 2.35 (95% CI 1.47–3.74) (Table 2) and 2.28 (95% CI 1.46–3.54) (Table 3), respectively. The H–L tests indicated a good fit for both models (*P* >.05).

**Table 2:** Multivariate analysis of CLR in association with postoperative delirium after hip fracture surgery using “totally adjusted” logistic regression model.

Variables	β	Standard error	OR (95% CI)	*P*
CLR (≥36.0 vs <36.0)	0.854	0.238	2.35 (1.47–3.74)	<.001
Albumin	-0.034	0.024	0.97 (0.92–1.01)	.153
RBC	0.282	0.236	1.33 (0.83–2.11)	.233
Sodium	0.050	0.031	1.05 (0.99–1.12)	.110
Sex (female vs male)	0.337	0.260	1.4 (0.84–2.33)	.195
Age (for each year increment)	0.040	0.016	1.04 (1.01–1.07)	.015
Fracture location (intertrochanteric vs femoral neck)	0.329	0.294	1.39 (0.78–2.47)	.263
ASA classification	–	–	–	.127
I	–	–	Reference	–
II	1.697	1.054	5.46 (0.69–43.06)	.107
III or more	1.935	1.051	6.93 (0.88–54.33)	.066
aCCI	–	–	–	.002
≤3	–	–	Reference	–
4	0.861	0.471	2.37 (0.94–5.96)	.068
5	1.257	0.502	3.51 (1.31–9.39)	.012
≥6	1.726	0.501	5.62 (2.1–15.01)	.001
Time from injury to operation (>72 vs ≤ 72 h)	0.725	0.239	2.07 (1.29–3.3)	.002
Operative method (fracture repair vs arthroplasty)	-0.198	0.319	0.82 (0.44–1.53)	.534
Surgical time (≥120 vs < 120 mins)	−0.270	0.269	0.76 (0.45–1.29)	.316

β, indicating regression coefficient.

aCCI = age-adjusted Charlson comorbidity index, ASA = American Society of Anesthesiologists, CI = confidence interval, CLR = high-sensitive C-responsive protein lymphocyte ratio, OR = odds ratio, RBC = red blood cell.

**Table 3:** Multivariate logistic regression for analysis of CLR in association with postoperative delirium after hip fracture surgery using stepwise backward elimination method.

Variables	β	Standard error	OR (95% CI)	*P*
CLR (≥ 36.0 vs <36.0)	0.822	0.226	2.28 (1.46–3.54)	<.001
Age (for each year increment)	0.053	0.016	1.05 (1.02–1.09)	.001
aCCI	–	–	–	<.001
≤3	–	–	Reference	–
4	0.839	0.465	2.31 (0.93–5.75)	.071
5	1.284	0.498	3.61 (1.36–9.59)	.010
≥ 6	1.775	0.489	5.9 (2.26–15.4)	<.001
Time from injury to operation (>72 vs ≤72 h)	0.783	0.227	2.19 (1.4–3.41)	.001

β, indicating regression coefficient.

aCCI = age-adjusted Charlson comorbidity index, CI = confidence interval, CLR = high-sensitive C-response protein lymphocyte ratio, OR = odds ratio, RBC = red blood cell.

## 4. Discussion

In this study, we identified the nonlinear relationship between hs-CLR levels and the incidence of postoperative delirium following hip fracture surgery in elderly patients. We also identified 2 clinically relevant thresholds, with hs-CLR values of 36.0 indicating a baseline risk and 86.0 denoting a threshold effect, and hs-CLR ≥ 36.0 was independently associated with an increased risk of delirium.

The fitted RCS curve illustrates a clear, consistent increase in the risk of delirium as hs-CLR levels rise, surpassing baseline risk at a threshold of 36.0, and culminating in a plateau effect at hs-CLR of 86.0. This finding underscores the potential utility of monitoring hs-CLR levels as a predictive biomarker for identifying patients at heightened risk of postoperative delirium. By establishing hs-CLR of 36.0 as a critical threshold for risk assessment, this study highlights the need for proactive intervention strategies aimed at mitigating delirium in this vulnerable population. Moreover, the ROC curve analysis corroborates this threshold with a similar cutoff value of 35.5, reinforcing the rationale for employing hs-CLR as a criterion to identify sensitive patient groups. The plateau effect at hs-CLR of 86.0 suggests a potential ceiling effect regarding the influence of inflammatory/immune processes on delirium process, likely suggesting that additional factors – such as psychological or environmental variables – could significantly contribute to the development of delirium.

The mechanisms linking hs-CLR levels to postoperative delirium in elderly hip fracture patients can be elucidated through several aspects. Elevated hs-CLR levels indicate a heightened inflammatory response that can disrupt neurotransmitter systems and promote neuroinflammation, leading to cognitive alterations and an increased risk of delirium in vulnerable populations.^[16]^ Additionally, these elevated levels may correlate with disruptions in the blood-brain barrier, allowing neurotoxic substances to enter the central nervous system and further exacerbate cognitive impairment.^[17]^ Chronic inflammation is also linked to immune dysregulation, diminishing the body’s ability to respond effectively to infections – common precipitants of postoperative delirium, such as pneumonia and urinary tract infections.^[18,19]^ Furthermore, elevated hs-CLR may reflect declining nutritional status, which is essential for cognitive health. Nutritional deficiencies can heighten the vulnerability of elderly patients to delirium, particularly under surgical stress.^[20]^

In the context of elderly hip fractures, CLR and its variants, along with other metrics, have shown promise in predicting mortality and morbidity. For instance, He et al^[21]^ utilized lymphocyte CRP score or a modified variant to predict 3-year mortality after surgery of intertrochanteric fractures, achieving C-statistics of 0.644 and 0.686, respectively. In their investigation, the authors implemented a scoring system that assigned values to different levels of CRP and lymphocyte counts, which differs from our approach of using a ratio-based method. Similarly, Zhu et al^[22]^ identified that a low lymphocyte-to-CRP ratio is associated with increased 1-year mortality, 30-day total complications, blood transfusion and extended length of stay in patients undergoing hemiarthroplasty for displaced femoral neck fracture. Up to this point, we are not aware of any research examining the relationship between hs-CLR and delirium, both within the context of hip fracture surgery and other medical or surgical domains. Our findings indicate that hs-CLR levels ≥36.0 are associated with a 2.28-fold increased risk of postoperative delirium following hip fracture surgery, and that the area under the curve of hs-CLR for predicting POD was approximately 0.62, indicating modest discrimination. Taken together, these findings suggest that hs-CLR may serve as a simple, readily available biomarker to help identify patients at higher risk of POD, but it should be used to supplement, rather than replace, clinical judgment and existing multifactorial risk assessment tools in elderly patients with hip fractures.

This study has several limitations that warrant consideration. First, its retrospective design inherently affects the accuracy of data collection and introduces selection bias, especially for comorbidities primarily self-reported by patients or their relatives. Second, the stringent inclusion and exclusion criteria may have resulted in either an overestimation or underestimation of the incidence. Nonetheless, this methodological rigor bolsters the internal validity of the observed association between hs-CLR levels and delirium. Furthermore, the incidence rates align with previously reported figures, reinforcing the credibility of our findings. Third, some critical variables, such as fluid intake, perioperative blood pressure fluctuations, preoperative mental status, sleep quality, and medication use, may influence the occurrence of delirium. However, due to the lack of available data on these factors, residual confounding remains a concern. Fourth, the study was conducted at a university-affiliated tertiary care facility designated as a Level Ⅰ trauma center, where patients may be with greater medical complexity or more severe fractures. Consequently, the generalizability of our findings necessitates validation in more diverse clinical settings or across broader populations.

In summary, this study elucidated the nonlinear dose-responserelationship between hs-CLR levels and the incidence of delirium, highlighting the independent risk associated with hs-CLR ≥36.0 in patients with hip fractures. These findings highlight the importance and potential of integrating hs-CLR into preoperative assessments, thereby enhancing preoperative risk counseling and supporting individualized risk management strategies.

## Acknowledgments

We are grateful to L.W. and Z.W. of Department of Orthpaedics Surgery for their kind help.

## Author contributions

**Conceptualization:** Yanbin Zhu, Yahui Zhang.

**Data curation:** Song Liu, Shanshan Zhang.

**Formal analysis:** Song Liu, Shanshan Zhang, Xiuting Li.

**Investigation:** Song Liu, Shanshan Zhang.

**Methodology:** Xiuting Li.

**Project administration:** Xiuting Li, Yanbin Zhu.

**Resources:** Yahui Zhang.

**Software:** Shanshan Zhang.

**Supervision:** Yahui Zhang.

**Writing – original draft:** Song Liu, Xiuting Li.

**Writing – review & editing:** Yanbin Zhu, Yahui Zhang.

## Supplementary Material


