# Supplementary material for: High-sensitivity C-reactive protein to lymphocyte ratio as a novel biomarker for predicting postoperative delirium in elderly patients with hip fractures: A retrospective cohort study
Source: Medicine (Baltimore). 2026 Feb 6;105(6):e47650. doi: 10.1097/MD.0000000000047650 (PMC12885664; doi:10.1097/MD.0000000000047650)

**Supplementary Figure 1**

Unadjusted RCS curve illustrating a significant nonlinear dose-response relationship between hs-CLR and the risk of delirium following surgeries in patients with hip fractures (n = 582), with the baseline risk point at hs-CLR of 36.0. The blue vertical dashed line marks the reference value of hs-CLR (36.0), and the black horizontal dashed line indicates an OR of 1.0.

RCS, Restricted cubic spline; hs-CLR, high-sensitivity C-reactive protein to lymphocyte ratio; OR, odds ratio; CI, confidence interval.


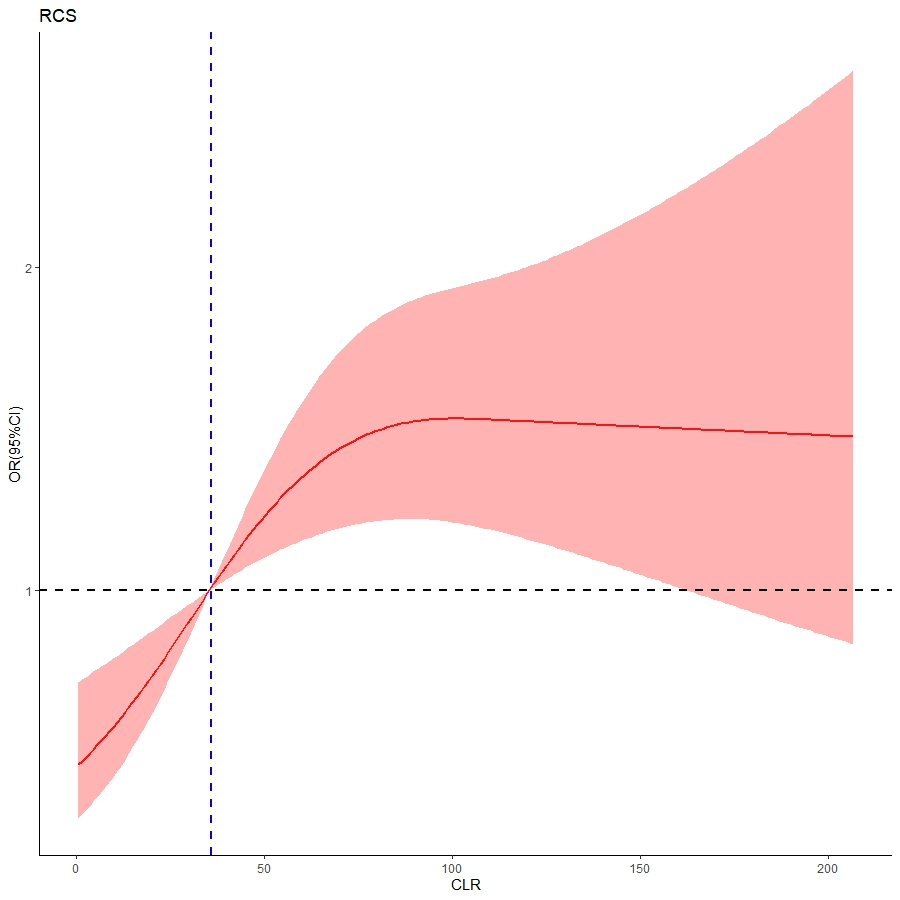

Supplement: Supplementary file 1 [file medi-105-e47650-s001.docx]
